# Antenatal and postnatal diagnoses of visible congenital malformations in a sub-Saharan African setting: a prospective multicenter cohort study

**DOI:** 10.1186/s12887-019-1831-x

**Published:** 2019-11-25

**Authors:** Igor Kamla, Nelly Kamgaing, Serge Billong, Joel Noutakdie Tochie, Paul Tolefac, Vincent de Paul Djientcheu

**Affiliations:** 1 Department of Surgery and sub-Specialties, Faculty of Medicine and Biomedical Science of University of Yaoundé I, Yaoundé, Cameroon; 2 Department of Pediatrics, Faculty of Medicine and Biomedical Science of University of Yaoundé I, Yaoundé, Cameroon; 3 Department of Pediatrics, University Hospital Centre, Yaoundé, Cameroon; 4 Department of Neurosurgery, Central Hospital, Yaoundé, Cameroon

**Keywords:** Congenital malformation, Epidemiology, Antenatal diagnosis, Yaoundé

## Abstract

**Background:**

Visible congenital malformations (VCMs) are one of the principal causes of disability in the world. Prenatal diagnosis is a paramount mandatory integral part of the follow up of pregnancies with VCM of the foetus in high-income setting. We aimed to determine the incidence of prenatal diagnosis of VCMs in a low-resource setting with no policy on antenatal diagnosis of VCMs.

**Methods:**

We carried out a prospective cohort multicenter study from July 2015 to June 2016 in 10 randomly selected maternity units of Yaoundé, Cameroon. We enrolled all newborns with one or more detectable VCMs at birth. Variables studied were findings of the 1st, 2nd and 3rd trimesters’ obstetrical ultrasound scans, in order to establish a concordance between the clinical and sonographic diagnoses of the VCMs and determine the frequency of antenatal diagnosis as well as the rate of medical abortion.

**Results:**

The incidence of VCMs was 9 per 1000 births. The main VCMs were malformations of the skeletal (4.3%), neurological (2.2%), and gastrointestinal (2.1%) systems. The sex ratio was 1.1. Among the malformed newborns, 37% were premature and in 18.5% the diagnosis of a VCM was confirmed after a therapeutic termination of pregnancy (following suggestive findings of a malformation on antenatal ultrasound scan). The prevalence of sonographic antenatal diagnosis of VCMs was 21%. Hydrocephalus was the most diagnosed VCM antenatally. The mean gestational age at which antenatal clinics were initiated was 15 ± 5 weeks. The mean number of obstetrical ultrasound scans performed was two.

**Conclusion:**

The incidence of VCMs in our resource-limited setting is high and antenatal diagnosis rates are very low. Overall, our study emphasizes on the importance antenatal diagnosis of VCMs, often overlooked in our setting. The goal being to reduce maternal and foetal morbidity in a setting already burdened by a high maternal and neonatal mortality.

## Background

Every year, more than 7.9 million children, or 6% of all neonates worldwide, are born with severe congenital disorders (neural tube defects, heart defects and trisomy 21) [1]. Due to limited antenatal diagnostic tests in sub-Saharan Africa (SSA), visible congenital malformations (VCMs) are easier to diagnose than occult congenital malformations (CMs) [2]. While some VCMs may be fatal due to incompatibility with life, others lead to permanent physical or mental handicaps. Hence, VCMs constitute occupational hazards of significant economic consent to any society. Furthermore, children growing with such handicaps often face psychological trauma due to peer or societal stigmatization [1]. To this end, the importance of antenatal diagnosis of VCMs in SSA, cannot be overemphasized. In high-income countries antenatal diagnosis of CMs is a mandatory follow up during pregnancy and there are a lot of sophisticated diagnostic tests for this purpose. Contrarily, in SSA, these CMs are mostly diagnosed by ultrasound scans done during pregnancy [2]. Though not yet a routine practice in these resource-limited settings, we cannot argue the merits of antenatal diagnosis of CMs, especially VCMs which are easier to diagnose. Thus, we proposed to conduct this study to determine the frequency of antenatal diagnosis of VCMs in a SSA setting.

## Methods

### Study design and setting

From July 2015 to June 2016, we conducted a prospective cohort study in 10 randomly selected maternity units of public health centres in Yaoundé, the capital city of Cameroon. These 10 maternity units were the maternity departments of six reference hospitals (the Gynaeco-Obstetrics and Pediatric Hospital, Yaounde General Hospital, Yaounde Central Hospital, Yaounde Military Hospital, University Hospital Center of Yaounde, and Hospital Center of Essos); the maternity departments of three district hospitals (District Hospital of Cité verte, Efoulan District Hospital, Biyem-Assi District Hospital) and lastly the maternity wards of two sub-divisional hospitals (Nkolndongo Social and Health Animation Center). Private hospitals were excluded.

### Study participants, study procedure and variables studied

After obtaining ethical approval and parental consents, we proceeded through clinical examination to enroll all living or dead newborns with one or more clinically detectable malformations (VCMs) as described below. Firstly, following delivery of the neonate by a midwife or an obstetrician, all VCMs were confirmed within an hour of delivery by a pediatrician who conducted a full neonatal physical examination including an auscultation of the heart for live neonates. Secondly, the mothers of the newborns delivered with VCMs were interviewed about their pregnancy follow up. Parents of stillborn neonates were interviewed after mourning the death of their neonate. The various trimesters of pregnancy were defined according to WHO, the 1^st^ trimester ranging from zero to 16th complete weeks; the 2^nd^ trimester from the 16th to 27th completed weeks and the 3^rd^ trimester from the 28th week to the 40th resolved week [3]. According to the Cameroon’s national protocol, drawn from recommendations of WHO, pregnant women are supposed to attend a minimum of 4 antenatal consultations (ANCs) with at least one ANC attended and at least one ultrasound scan done in each trimester [3]. Since 2009, Cameroon adopted the consensus of the French College of Foetal Ultrasound for a good screening for prenatal malformations which recommends that ultrasound scans should be performed at the 12^th^, 22^nd^ and 32^nd^ weeks of gestational [3]. This corresponds to at least one sonographic scan performed each trimester. Besides this minimum number of ANCs and ultrasound scans recommended, in conformity with the guidelines of the Cameroonian Society of Gynaecologists and Obstetricians, it is routine practice for obstetricians to counsel pregnant women to come for an ANC and/or have a sonographic scan whenever they are ill, have decreased or absent foetal kicks, or a new unusual pregnancy symptom or sign. It is worth to mention that the main antenatal diagnostic test used in Cameroon is ultrasound scan and other tests such as amniocentesis or genetic diagnosis are not yet available [2]. The National Council of Cameroonian Medical Doctors recommends only medical abortions, also called therapeutic termination of pregnancy defined as a medical act to arrest a pregnancy irrespective of its gestational age when its continuation poses a threat to maternal life or when a congenital fœtal malformation incompatible with life is detected antenatally. Any termination of a pregnancy performed for other purposes is considered illegal in Cameroon. The parents were interviewed on the findings of these three obstetrical ultrasound scans done in each of the trimesters, in order to establish a concordance between the clinical and sonographic diagnoses of the VCMs. We also studied the age of onset of ANC, the number of ANCs attended and who performed the ultrasound scans.

### Data analysis

We calculated the incidence of VCMs, their frequency of antenatal diagnosis and the rate of medical abortions. The threshold of statistical significance was set at 5%.

## Results

### Incidence of visible congenital malformations

**Fig. 1:**
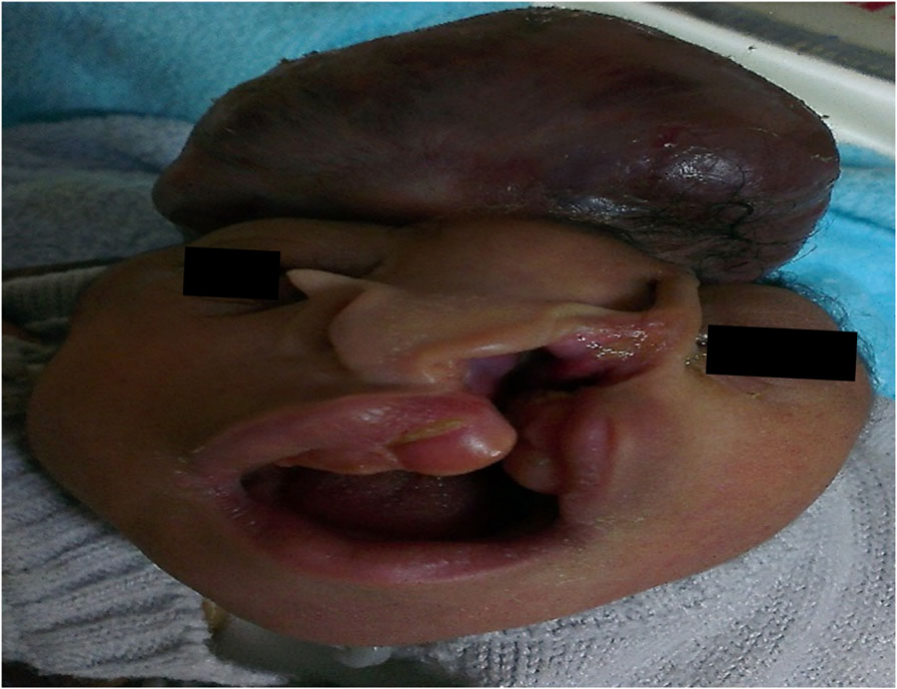
Encephalocele, cleft lip and palate. Male newborn at Biyem-Assi District Hospital at 34 weeks of gestation weighing 2000 g with encephalocele associated with a left labio-palatal cleft

**Fig. 2:**
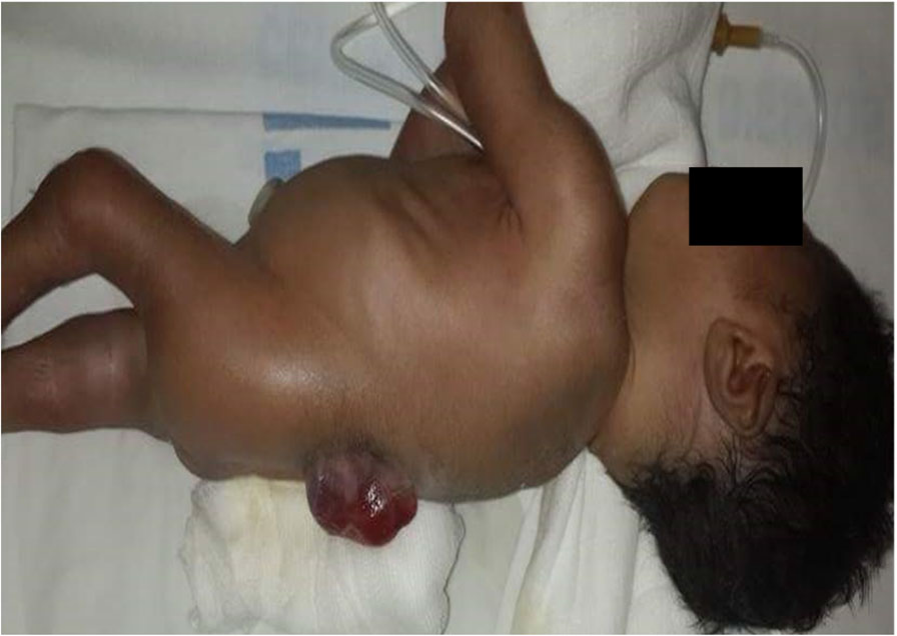
Myelomeningocele in the lumbosacral region. Newborn male, born at Essos Hospital Center in Yaounde at 32 weeks of gestation, weighing 1700 g with head circumference of 43 cm and presenting a myelomeningocele, complicated with hydrocephalus, and amyotrophy of lower limbs

We identified 189 cases of VCMs out of a total of 21,113 births, an overall incidence of 9 cases per 1000 births. These malformed neonates were delivered by 188 mothers. The highest incidence was observed at the Yaoundé Gynaeco-Obstetrics and Pediatric Hospital, 2.3 per 1000 births (Table 1). The main VCMs were malformations of the skeletal (4.3%), neurological (2.2%), and gastrointestinal (2.1%) systems (Table 2). The most common types were (incidence per 1000 births): polydactyly (2.1), club feet (1.1), neural tube defects (NTD) (1.4), hydrocrocephalus (1.1), cleft lip and palate (0.5) (Fig. 1), omphalocele (0.5), sexual ambiguity (0.5) and facial dysmorphism (0.4). A single malformation was observed in 70.4% of neonates with malformations, whereas a polymalformation syndrome was seen in 29.6% of newborns (Table 3). The association of myelomeningocele (Fig. 2), hydrocephalus and/or lower limb abnormality was the most common polymalformation (17.9%).

**Table 1:** Division of malformed babies according to maternity origin

Maternity units	Annual number of births	Number of malformed newborns	Incidence per 1000 births
Central Hospital of Yaoundé^a^	3496	28	1.3
Gynaeco-obstetrics and Pediatric Hospital of Yaounde^a^	3163	49	2.3
General Hospital of Yaoundé^a^	792	8	0.4
University Hospital Center of Yaoundé^a^	850	4	0.2
Military Hospital of Yaoundé^a^	604	8	0.4
Biyem-Assi District Hospital	2730	12	0.6
Efoulan District Hospital	1586	14	0.7
Nkolndongo Social and Health Animation Center	4056	29	1.4
Hospital Center of Essos	2233	15	0.7
District Hospital of Cité Verte	1603	22	1
Total	21,113	189	9

^a^University Teaching Hospitals

**Table 2:** Distribution of visible malformations according to the international of Diseases (ICD, 2012), and according to their isolated nature and/or associated with other malformations

Types of malformations	Code ICD	Number	Isoleted cases N (%)	Associated cases N (%)	Incidence/1000 births
SKELETAL MALFORMATIONS (91)					4.3
Polydactyly	Q69	45	38 (84.4)	7 (15.6)	2.1
Club foot	Q66.0	24	12 (50)	12 (50)	1.1
Micromelia		6	0 (0)	6 (100)	
Macrocephaly	Q75.3	4	0 (0)	4 (100)	
Muscular atrophy of the lower limbs		3	0 (0)	3 (100)	
ectrodactyly	Q71.6	3	2 (66.7)	1 (33.3)	
Varus feet		3	3 (100)	0 (0)	
Ankylosis of the knee		3	1 (33.3)	2 (66.7)	
Syndactyly	Q70	2	2 (100)	0 (0)	
Agenesis of fingers or toes		2	0 (0)	2 (100)	
Convex foot		1	1 (100)	0 (0)	
Phocomelia	Q73.1	1	0 (0)	1 (100)	
MALFORMATIONS OF CNS* (46)					2.2
*NTD*		29			1.4
Myelomeningocel	Q05	16	6 (37.5)	10 (62.5)	
Meningocel	Q05	1	0 (0)	1 (100)	
Anencephaly	Q00.0	8	8 (100)	0 (0)	
Encephalocel	Q01	4	3 (75)	1 (25)	
*HYDROCEPHALUS*	Q03	24	13 (54.2)	11 (45.8)	1.1
*MICROCEPHALIA*	Q02	4	2 (50)	2 (50)	
GASTROINTESTINAL MALFORMATIONS (44)					2.1
*OROFACIAL CLEFT*		18			0.9
Cleft lip	Q36	6	6 (100)	0 (0)	
Labio-palatal cleft	Q37	11	7 (63.6)	4 (36.4)	
Palatal cleft	Q35	1	1 (100)	0 (0)	
*ABDOMINAL PARIETAL DEFECT*		15			0.7
Omphalocele	Q79.2	10	5(50)	5(50)	
Gastroschisis	Q79.3	5	5(100)	0(0)	
*OTHER DIGESTIVES MALFORMATIONS*					
Imperforate anal	Q42.3	6	4 (66.7)	2 (33.3)	
Macroglossia	Q38.2	5	0(0)	5 (100)	
Salivary froglet	K11.6	1	1 (100)	0 (0)	
UROGENITAL MALFORMATIONS (25)					1.2
Sexual ambiguity	Q56.4	11	7 (63.3)	4 (36.7)	
Hypospadias	Q54	6	6 (100)	0(0)	
Absence of external genitalia		4	0 (0)	4 (100)	
EYE, EAR, FACE AND NECK MALFORMATIONS (24)					1.1
Facial dysmorphism	Q67.0	9	2 (22.2)	7 (77.8)	
Low implanted ears	Q17.4	5	1 (20)	4 (80)	
Arrhinia		3	0 (0)	3 (100)	
Anophthalmos	Q11	2	0 (0)	2 (100)	
Hypotelorism		3	1 (33.3)	2 (66.7)	
Proboscis		2	0 (0)	2(100)	
Synophthalmia		1	0 (0)	1 (100)	
Exophthalmos		1	1 (100)	0 (0)	
Cervical teratomas		1	1 (100)	0 (0)	
Eversions of the eyelids		1	1 (100)	0 (0)	
VASCULAR MALFORMATIONS (5)		5			0.2
Lymphangioma	D18.1	4	4 (100)	0 (0)	
Hemangioma	D18.04	1	1 (100)	0 (0)	

*CNS* Central Nervous System, *NTD* Neural tube defects

**Table 3:** Distribution of the principal polymalformations identified

Polymalformations	Number	Incidence per 1000 births
Myelomeningocele + hydrocephalus	5	2.4
Myelomeningocele + hydrocephalus + abnormalities of the lower limbs^a^	5	2.4
Thanatophoric dwarfism^b^	5	2.4
Beckwith Wiedemann syndrome^c^	4	1.9
Polydactylies + club feet	3	1.4
Trisomy 21^d^	3	1.4
Sirenomelia (baby mermaid)^e^	2	0.9
Prune Belly Sequence^f^	2	0.9
labio-palatal cleft + Neural tube defect	2	0.9
Major aplasia of the ear^g^	2	0.9
Hydrocephalus + clubfoot	2	0.9
Sexual ambiguities + limb deformity	2	0.9
VACTERL association^h^	2	0.9
Cyclopia^i^	1	0.5
Labio-palatal cleft + microcephaly	1	0.5
Hydrocephalus + Labio-palatal cleft + phocomelia	1	0.5
Hydrocephalus + Sexual ambiguities	1	0.5
Thoracoabdominal ectopia^j^	1	0.5
Achondroplasia^k^	1	0.5
Ethmocephaly^l^	1	0.5
Potter sequence^m^	1	0.5
Anophthalmia + arhinia + astoma	1	0.5
Arthrogryposis syndrome^n^	1	0.5
Trisomy 18^o^	1	0.5
Other polymalformations	6	
Total	56	

^a^Muscular atrophy of the lower limbs and/or feet

^b^Macrocephaly + prominent abdomen + micromelia + narrow chest

^c^Omphalocele + macroglossia + gigantism

^d^Facial dysmorphism + low implanted ears + short and wide neck + single palmar fold

^e^Absence of external genitalia + anal imperforation + fusion of the lower limbs

^f^Abdomen of batrachian + bladder

^g^Severe hypoplasia of the ear flag + anomaly of the external auditory canal

^h^Anal imperforation + joint stiffness or club foot + atresia of the esophagus

^i^Microcephaly + synophthalmia + arhinia + proboscis

^j^Lack of midline closure with thoracic and abdominal evisceration

^k^Macrocephaly + prominent abdomen + rhizomic micromelia + narrow chest

^l^Hypotelorism + arhiny + proboscis

^m^Facial dysmorphism + joint stiffness in the context of prolonged oligoamniosis

^n^Clubfoot + stiffness of the knees and other joints

^o^Facial dysmorphism + low implanted ears + closed fists + finger overlap

### Characteristics of malformed newborns

Out of the 189 newborns, 14 were delivered from a twin pregnancy with both twins malformed. Thirty-seven percent (37%) of malformed neonates were born premature. The sex ratio was 1.1. Eight babies were of unknown gender either because it did not exist at all or because it was not differentiable (suspected cases of sexual ambiguities). Thirty-nine neonates (20.6%) were born via caesarean section. The main indication of caesarean delivery was cephalo-pelvic disproportions (52.3%). The causes of these disproportions were mainly due to macrosomia, macrocrania, and foetal malpositions. There were 10 intra-uterine foetal deaths (IUFD) and five intrapartum deaths; the main causes were polymalformations and severe malformations such as gastroschisis. The average weight of neonates with VCMs was 2700 ± 1000 g with extremes ranging from 400 g to 5000 g. More than one-third (36.5%) of these newborns had low birth weights (less than 2500 g). Sixty six percent (56%) of the ultrasound scans were performed by sonographer technician, 25% by a radiologist and 19% were done by an obstetrician.

### Maternal characteristics

Mean maternal age was 27.4 ± 5.7 years with extremes ranging from 15 to 43 years. Almost half of the mothers (49.5%) had an age between 26 and 35 years old. The average paternal age was 34 ± 7.2 years with extremes ranging from 19 to 54 years. More than half of the mothers had a tertiary level of education (56.4%), 28.7% were housewives and 61.7% were single. More than one-third (36.2%) of the mothers of malformed newborns were primiparous and 29.3% were primigravidae.

### Antenatal diagnosis according to the type of malformation

The average number of ANCs attended by mothers was 4.6 ± 1.9 (range: 1 to 10). One hundred and seventy-nine (95.2%) had attended at least one ANC and 28.5% of the mothers attended less than 4 ANCs, which is the minimum recommended by WHO and adopted in Cameroon. The mean gestational age at onset of ANC was 15 ± 5 weeks with extremes ranging from 6 to 32 weeks. More than one-third (36.3%) of the mothers who attended ANC started after the first trimester (Table 4).

**Table 4:** Distribution according to the trimester of the start of ANC

Start of ANC (*n* = 179)	Numbers	Percentages (%)
1st trimester	114	63.7
2nd trimester	60	33.5
3rd trimester	5	2.8

*ANC* Antenatal consultation

At least one antenatal ultrasound scan was done by 176 (93%) mothers of neonates with VCMs, including 42.3% in the 1^st^ trimester, 77.1% in the 2^nd^ trimester and 75.4% in the 3^rd^ trimester. The mean gestational age at which sonographic scans were performed in each trimester was 11, 23 and 33 weeks for the first, second and third trimesters respectively. The average number of ultrasounds was 2 with extremes ranging from 1 to 5. Thus, an antenatal presumptive diagnosis was evoked in 44 newborns with VCMs, the majority of them in the third trimester. However, a concordance between this ultrasound diagnosis and the clinical diagnosis was established in 37 of the 44 neonates, that is an antenatal diagnosis rate of 21%.

Thirty-five (18.5%) of the 189 newborns with VCMs were delivered via therapeutic termination of prgnancy (TTP) after a presumptive VCMS diagnosis was made on antenatal ultrasound. The TTP was performed when a malformation incompatible with live was diagnosed antenatally or when a fetus with a potentially surgical corrected malformation which could not await term delivery; as was the case two fetuses with severe bilateral urethro-vesico-uretero-hydronephrosis (Brune Belley Syndrome), a fetus with hydrocephalus, one with hydronephrosis plus hypospadia and one with omphalocele.

Visible central nervous systems (CNS) malformations including hydrocephalus, was the most diagnosed malformations in the antenatal group because 17 out of 24 cases were diagnosed among the 22 who performed an antenatal ultrasound. Visible CNS malformations were followed in order of frequency by gastrointestinal and skeletal VCMs (Table 5). Regarding neural tube defects (NTD), the antenatal diagnosis of anencephaly was made in 50% of cases and all diagnosed cases benefited from a TTP. That of spina bifida was 16.6%. No cases of orofacial clefts, polydactyly and club feet were diagnosed antenatally.

**Table 5:** Rates of antenatal diagnosis and medical termination of pregnancy per type of malformation

Types of visual congenital malformations (VCMs)	Total	Antenatal ultrasound performed	Antenatal diagnosis N (%)	Therapeutic termination of Pregnancy N (%)
CENTRAL NERVOUS SYSTEM				
Hydrocephalus	24	22	17 (70.8)	15 (62.5)
Anencephaly	8	7	4 (50)	4 (50)
Spina Bifida	17	12	3 (16.6)	2 (11.8)
Microcephaly	4	4	2 (50)	2 (50)
GASTROINTESTINAL SYSTEM				
Gastroschisis	5	5	2 (40)	2 (40)
Omphalocele	10	8	3 (30)	2 (20)
VASCULAR SYSTEM				
Cystic lymphangioma	4	3	1 (25)	1(25)
EYE, EAR, FACE AND NECK				
Hypotelorism	3	2	1 (33.3)	(33.3)
Cyclopia	1	1	1 (100)	1 (100)
SKELETAL SYSTEM				
Thanatophoric dwarfism	5	5	2 (40)	2 (40)
Achondroplasia	1	1	1 (100)	1 (100)
URO-GENITAL SYSTEM				
Prune Belly Sequence	2	2	2 (100)	2 (100)

## Discussion

This prospective multicenter cohort study aimed to determine the incidence of antenatal diagnosis of VCMs in Yaoundé, a resource-limited setting of SSA. We found that the incidence of VCMs was 9 per 1000 births. Among the malformed newborns, 37% were premature and 18.5% were confirmed after a TTP following an antenatal diagnosis of a VCM. The prevalence of sonographic antenatal diagnosis of VCMs was 21% and hydrocephalus was the most diagnosed VCMs. The mean gestational age at which ANCs were initiated was 15 ± 5 weeks.

The overall incidence of VCMs in our study was 9 cases per 1000 births. This incidence is higher than that of the multicenter study carried out in the Democratic Republic of Congo in 2012 which found an incidence of VCMs of 6.7 newborns per 1000 births [4]. We observed the highest frequency (25.9%) of newborns with VCMs at the Gynaeco-Obstetrics and Pediatric Hospital of Yaoundé. This may be due to the fact that it is one of the main referral obstetrics and pediatrics hospital in Yaoundé and its environs. Hence, the Gynaeco-Obstetrics and Pediatric Hospital of Yaoundé is more likely to receive a lot referrals of VCMs detected during pregnancy and after delivery from other less equipped hospitals. This hospital is also more likely to be referred more cases of neonates with VCMs requiring surgical care due to the fact that it has more pediatric surgeons and a better surgical infrastructure for the management of VCMs requiring surgical interventions compared to other hospitals in Yaoundé. Moreover, these results are also certainly influenced by the fact that the obstetrical and pediatric staff of the hospital had recently undertaken a refresher course on the recognition of CMs at birth geared at their timely diagnosis and management.

The most common VCMs were: skeletal (4.3%), neurological (2.2%), and gastrointestinal (2.1%) and urogenital (1.2%) malformations. This order of frequency per system is closed to that found by several authors [5–7]. Polymalformations were observed in 29.6% of VCMs. This prevalence rate is lower than those observed in Abidjan [8], Paris [9] and Belgium [10] which were 6.2, 3.7 and 3.3 per 1000 births, respectively. This disparity in prevalence rates may be due to under-reporting of VCMs which still seems to go unnoticed in at birth and the neonatal period in many settings.

In this study, the sole antenatal diagnostic test was obstetrical ultrasound scan. According to the WHO recommendations, at least three ultrasounds should be performed during pregnancy, one per trimester [3]. These scans aim to ensure foetal well-being, and the absence of CMs. This antenatal diagnosis is important to pregnant women because while some choose the continuation of their gestation, others seek a TTP. Of the 176 neonates who had at least one antenatal ultrasound, a sonographic-clinical match was only established in 37, an antenatal diagnosis rate of 21%. This low sonographic-clinical correspondence was observed for instance in case of “holoproencephaly” on antenatal ultrasound scans, but the presence of “myelomeningocele + hydrocephalus + cleft lip-palate” at birth; or in case of “gastrointestinal visible malformation” on ultrasound, but the presence of “agenesis of the abdominal wall with exposure of the viscera + absence of external genital organs + lumbosacral agenesis and lower limbs” at birth. This antenatal diagnosis makes it possible to terminate the pregnancy if the VCM seen on antenatal ultrasound is deemed incompatible with life, or if emergency management is necessary to improve the vital prognosis of the foetus. It is worth to mention that our prevalence rate of antenatal diagnosis was higher than the 16.3% found at the Douala General Hospital, Douala, Cameroon of in the year 2012 [2]. This discrepancy in prevalence rates could be explained by the fact that we included several health facilities specialized mainly in maternal and child health in Yaoundé compared to the Douala General Hospital which is not only specialized in maternal and child health. The frequency of antenatal diagnosis in our context is far below the 69.1% obtained in France [9]. The low rate of antenatal diagnosis in the present study could be explained by an insufficient number of prenatal ultrasound scans performed to pregnant women especially in the 2nd and 3rd trimester where VCMs are easily detectable and the fact that more than half (56%) of the sonographic scans were performed my ultrasound technicians who have are trained on elementary sonography such as pregnancy detection, foetal sex identification and rarely detection of CMs in our setting. We found that anencephaly had an antenatal diagnosis rate of 50 and 40% for gastroschisis. But in Europe, the antenatal diagnosis of anencephaly is made in 100% of cases since the twenty-first century, because this malformation is frequent and incompatible with life, no child should be born with this anomaly [9]. The antenatal diagnosis of gastroschisis was 98% in Paris [9]. It is therefore urgent to train all personnel performing an antenatal ultrasound scan because the sensitivity of the ultrasound depends greatly on the operator’s qualification [3]. Some malformations diagnosed prenatally that did not benefit from TTP were related to the family’s refusal.

According to the recommendations of the French College of Fetal Ultrasound for a good screening for prenatal malformations, ultrasounds should be performed at the 12^th^, 22^nd^ and 32^nd^ week of gestational amenorrhea, with an optimal age between 21 and 23 week of gestational [3]. However, we found that antenatal ultrasounds, especially those of the 2^nd^ and 3^rd^ trimesters, were not performed at the right time, which could compound, contribute to the low antenatal diagnosis rate. More than one-third (36.3%) of mothers who delivered newborns with VCMs started ANCs after the first trimester of pregnancy. As a result, abnormalities of foetal morphology or nuchal translucency were not timely diagnosed antenatally, hindering adequate pregnancy follow-up and anticipation of TTP or timely surgical management of newborns with VCMs requiring surgery.

**Fig. 3:**
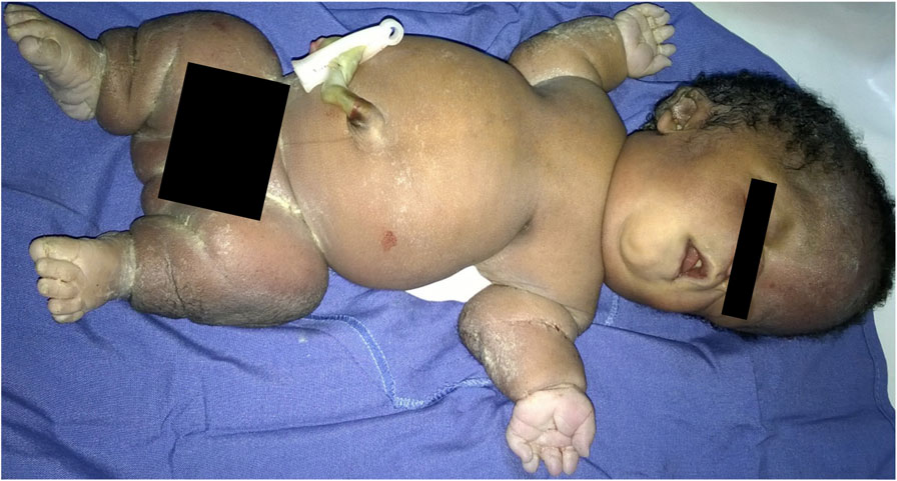
Thanatophoric dwarfism. Male neonate born at the Gynaeco-obstetrics and Pediatric Hospital of Yaoundé at 39 weeks of gestation weighing 3800 g, with a head circumference 41 cm and presenting thanatophoric dwarfism syndrome that combines micromelia, macrocrania with facial dysmorphism, prominent abdomen and narrow chest. It is a genetically based malformation incompatible with life

**Fig. 4:**
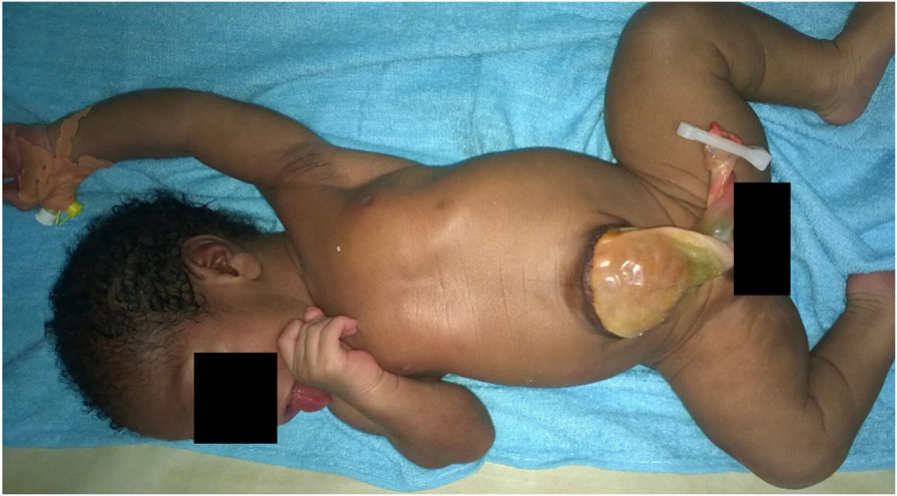
Beckwith Wiedemann Syndrome. Male newborn delivered at the Gynaeco-obstetrics and Pediatric Hospital of Yaoundé at 41 *weeks of gestation*, and presenting with Beckwith Wiedemann syndrome which associates omphalocele, macroglossia and gigantism (size = 53 cm, weight = 4600 g)

Fifteen newborns with VCMs were delivered stillborn, with majority of the deaths occurring intra-uterinelly. This result suggest the role of VCMs in neonatal mortality, which is one of the leading cause of neonatal mortality [1, 11–13]. The main indication for caesarean section in our study was cephalo-pelvic disproportionation related to macrocranium due to either thanatophoric dwarfism (Fig. 3), hydrocephalus or Beckwith Wiedemann syndrome (Fig. 4).

We acknowledge the following limitations of the present study. Firstly, the diagnosis of VCMs was made antenatally with ultrasound scans and at birth through clinical examination of the newborn without in-depth investigations of probably associated occult CMs through cardiac ultrasound, abdominopelvic ultrasound scan, spinal x-ray, magenetic resonance imaging or more sophisticated tests like amniocentesis and chorionic villi sampling for genetic tests. Although this was not the scope of our study, it is likey that we may have overlooked some associated and even later fatal neonatal occult malformations in newborns presenting with a visible single malformation or polymalformation syndrome. The inability to carry out the aforementioned imaging and genetic tests due to their relatively expensive cost and invailability in our resource-challenged setting was a significant compounding factor to this shortcoming. Public health authorities’ integration (and subsidization of the price) of an algorithm for antenatal screening of VCMs in our context could significantly reduce the under-reporting of VCMs in our setting and go a long way to terminate VCMs incompatible with life during pregnancy or help anticipate the neonatal care of neonates with VCMs requiring specific treatment and attention at birth. However, through a cohort design we have contributed in providing data on the scarcity of both antenatal and postnatal diagnoses of VCMs in the tropics.

## Conclusion

Visible congenital malformations are common in Yaoundé. The frequency of antenatal diagnosis is still very low due to an insufficient number of antenatal ultrasound scans performed and an inability to make the diagnosis during the realization of these ultrasound scans. Some major malformations diagnosed prenatally are not always followed by a therapeutic termination of pregnancy. It is therefore crucial to sensitize women to consult early in case of suspicion of pregnancy and to have obstetrical ultrasounds performed by qualified health care providers. The creation of a national registry of visible congenital malformations could become a sentinel and allow to consider a strategy of antenatal diagnosis, genetic counseling and prevention of these malformations.

## Data Availability

The datasets used and/or analyzed during the current study are available from the corresponding author on reasonable request.

## Abbreviations

ANC: Antenatal consultations
CMs: Congenital malformations
ICD: International of Diseases
IUFD: In-utero foetal deaths
NTD: Neural tube defects
TTP: Therapeutic termination of pregnancy
VCMs: Visible congenital malformations

## References

[1] Organisation mondiale de la santé. SOIXANTE-TROISIÈME ASSEMBLÉE MONDIALE DE LA SANTE: Malformations congénitales. [Accessed 10 August 2019]. Available at: http://apps.who.int/gb/ebwha/pdf_files/WHA63/A63_10-fr.pdf

[2] Tchente Nguefack C, Aurore ND, Charlotte B, Esther B, Eugene BP (2015). Prenatal diagnosis of congenital malformations in Douala general hospital. Open J Obstet Gynecol.

[3] Collège français d’échographie fœtale (2006). 10e symposium d’imagerie pédiatrique et périnatale.

[4] Lubala TK, Shongo MY, Munkana AN, Mutombo AM, Mbuyi SM, Momat FK (2012). Malformations congénitales à Lubumbashi (République Démocratique du Congo): à propos de 72 cas observés et plaidoyer en faveur du développement d’un Registre National des Malformations Congénitales et d’un Centre National de Référence de Génétique Humaine. Pan Afr Med J.

[5] Kouame BD, N’guetta-Brou IA, Kouame GSY, Sounkere M, Koffi M, Yaokreh JB (2015). Epidemiology of congenital abnormalities in West Africa: results of a descriptive study in teaching hospitals in Abidjan: cote d’Ivoire. Afr J Paediatr Surg AJPS.

[6] Fiogbe MA, Goudjo E, Gbenou AS, Fiogbe DA, Tonato-Bagnan AJ (2013). Les malformations congénitales cliniquement visibles et facteurs de risque répertoriés chez les nouveau-nés à Cotonou. J Rech Sci Univ Lome.

[7] Tayebi N, Yazdani K, Naghshin N (2010). The prevalence of congenital malformations and its correlation with consanguineous marriages. Oman Med J.

[8] Amon-Tanoh-Dick F, Gouli JC, N’gouan-Doumoua AM, Aka J, Napon-Kini H (2006). Epidémiologie et devenir immédiat de malformations du nouveau-né au CHU de Yopougon Abidjan. Côte d’Ivoire.

[9] Vigan CD, Khoshnood B, Lhomme A, Vodovar V, Goujard J, Goffinet F (2008). Prévalence et diagnostic prénatal des malformations en population parisienne. Data Rev.

[10] Gillerot Y, Mols M. Quinze années de surveillance des malformations congénitales dans le Hainaut et dans la province de Namur: Enseignements et recommandations. Services publics de Wallonie. 2009:1–50.

[11] Ndombo PK, Ekei QM, Tochie JN, Temgoua MN, Angong FTE, Ntock FN, Mbuagbaw L (2017). A cohort analysis of neonatal hospital mortality rate and predictors of neonatal mortality in a sub-urban hospital of Cameroon. Ital J Pediatr.

[12] Tochie JN, Choukem S-P, Langmia RN, Barla E, Koki-Ndombo P (2016). Neonatal respiratory distress in a reference neonatal unit in Cameroon: an analysis of prevalence, predictors, etiologies and outcomes. Pan Afr Med J.

[13] Mbonda A, Endomba FT, Kanmounye US, Nkeck JR, Tochie JN (2019). Diagnosis of Fraser syndrome missed out until the age of six months old in a low-resource setting: a case report. BMC Pediatr.

